# Combined Treatment With Environmental Enrichment and (-)-Epigallocatechin-3-Gallate Ameliorates Learning Deficits and Hippocampal Alterations in a Mouse Model of Down Syndrome

**DOI:** 10.1523/ENEURO.0103-16.2016

**Published:** 2016-11-08

**Authors:** Silvina Catuara-Solarz, Jose Espinosa-Carrasco, Ionas Erb, Klaus Langohr, Juan Ramon Gonzalez, Cedric Notredame, Mara Dierssen

**Affiliations:** 1 Cellular and Systems Neurobiology, Systems Biology Program, the Barcelona Institute of Science and Technology, Centre for Genomic Regulation (CRG), 08003, Barcelona, Spain; 2 Universitat Pompeu Fabra (UPF), Barcelona, 08003, Spain; 3 Human Pharmacology and Clinical Neurosciences Research Group, Neurosciences Research Program, Hospital Del Mar Medical Research Institute (IMIM), Barcelona, 08003, Spain; 4 Comparative Bioinformatics, Bioinformatics, and Genomics Program, Barcelona Institute of Science and Technology, Centre for Genomic Regulation, Barcelona, 08003, Spain; 5 Department of Statistics and Operations Research, Universitat Politècnica De Catalunya/BarcelonaTech, Barcelona, 08034, Spain; 6 Centre for Research in Environmental Epidemiology, Barcelona, 08003, Spain; 7 Centro De Investigación Biomédica En Red De Epidemiología Y Salud Pública, Madrid, 28029, Spain; 8 Centro De Investigación Biomédica En Red De Enfermedades Raras, Madrid, 28029, Spain

**Keywords:** (-)-epigallocatechin-3-gallate, Down syndrome, environmental enrichment, excitation-inhibition imbalance, neuroplasticity

## Abstract

Intellectual disability in Down syndrome (DS) is accompanied by altered neuro-architecture, deficient synaptic plasticity, and excitation-inhibition imbalance in critical brain regions for learning and memory. Recently, we have demonstrated beneficial effects of a combined treatment with green tea extract containing (-)-epigallocatechin-3-gallate (EGCG) and cognitive stimulation in young adult DS individuals. Although we could reproduce the cognitive-enhancing effects in mouse models, the underlying mechanisms of these beneficial effects are unknown. Here, we explored the effects of a combined therapy with environmental enrichment (EE) and EGCG in the Ts65Dn mouse model of DS at young age. Our results show that combined EE-EGCG treatment improved corticohippocampal-dependent learning and memory. Cognitive improvements were accompanied by a rescue of cornu ammonis 1 (CA1) dendritic spine density and a normalization of the proportion of excitatory and inhibitory synaptic markers in CA1 and dentate gyrus.

## Visual Abstract

**Figure:**
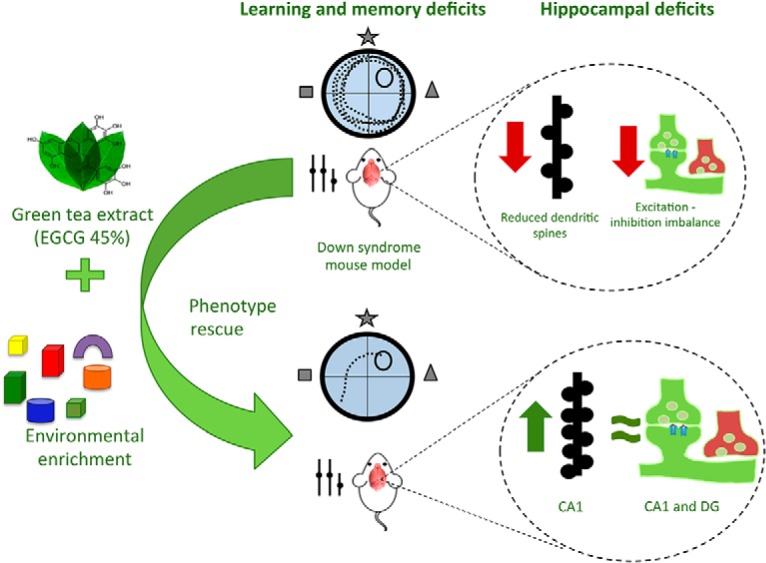

## Significance Statement

Therapeutic methods for improving intellectual disability in Down syndrome (DS) are limited, and their outcome remains unsatisfactory. Recently, we demonstrated that combined treatment with (-)-epigallocatechin-3-gallate (EGCG) and cognitive stimulation rescued cognitive deficits in DS individuals in a phase II clinical trial and also in a middle-age Ts65Dn mouse model of DS. Here, we show that environmental enrichment (EE) plus EGCG treatment improves corticohippocampal-dependent learning and memory deficits in young trisomic mice, restores cornu ammonis 1 (CA1) hippocampal dendritic spine density, and mitigates disruptions in excitatory/inhibitory synaptic puncta in CA1 and dentate gyrus. Our results suggest that therapies with the capacity to simultaneously target several abnormal processes underlying intellectual disability and use physiologic plasticity-enhancing interventions such as EE are optimal for disease-modifying interventions.

## Introduction

Down syndrome (DS) is the most common genetic form of intellectual disability, with ∼10 in 10,000 and 14 in 10,000 live births in European countries (Khoshnood et al., 2011) and the United States (Parker et al., 2010), respectively. It arises from the presence of an extra copy (or major portion) of chromosome 21 (Hsa21), leading to a complex genetic imbalance (Antonarakis et al., 2004). Individuals with DS show moderate to severe cognitive impairment, with an average intellectual quotient of 40–50 (de Sola et al., 2015) and 39.4% in the mild intellectual disability range of 50–70. The neuropsychological profile is characterized by marked hippocampal-dependent deficits particularly affecting spatial learning, memory, and executive functions, among other cognitive domains (Chapman and Hesketh, 2000; Nadel, 2003; Pennington et al., 2003). These cognitive deficits are associated with distinct neuro-architectural, synaptic, and neurochemical alterations (Lott and Dierssen, 2010; Dierssen, 2012). At the cellular level, there is a reduction in dendritic number and complexity in cortical and hippocampal neurons, which affects synaptic connectivity (Becker et al., 1986). Furthermore, accumulating evidence suggest that DS pathophysiology is tightly associated with a disruption of the balance between the excitatory and inhibitory neuronal systems (Reynolds and Warner, 1988; Risser et al., 1997; Seidl et al., 2001; Bhattacharyya et al., 2009). These abnormalities are of particular importance because they are related to disruptions in neural plasticity, which is essential for cognition (Baroncelli et al., 2011).

Several research groups have shown that it is possible to partially rescue DS phenotypes using nonpharmacological strategies such as postnatal handling or cognitive training by environmental enrichment (EE) that ameliorate behavioral and brain alterations in the Ts65Dn mouse model of DS (Martínez-Cué et al., 2002; Dierssen, 2003; Begenisic et al., 2011; Chakrabarti et al., 2011; Golabek et al., 2011). It is widely accepted that EE is a cognitive enhancing intervention that promotes synaptic plasticity, adult neurogenesis, and epigenetic modifications, among other processes (Sale et al., 2014). However, despite its beneficial effects, EE is not sufficient to promote long-lasting dendritic spine remodeling in Ts65Dn mice (Dierssen, 2003) or significant developmental changes in DS children (Mahoney et al., 2004).

More recently, (-)-epigallocatechin-3-gallate (EGCG), the most abundant catechin found in green tea, with antioxidant and neuroprotective properties, has been shown to efficiently improve cognitive phenotypes in DS individuals and mouse models (De la Torre et al., 2014), ameliorate synaptic plasticity impairment *in vitro* (Xie et al., 2008), and restore excitatory/inhibitory (E/I) imbalance in Ts65Dn mice (Souchet et al., 2015). EGCG is a natural inhibitor of the kinase activity of Hsa21 candidate gene Dyrk1A (Bain et al., 2003), whose overexpression is sufficient to induce cognitive and neuromorphologic alterations (Altafaj et al., 2001; Martinez de Lagran et al., 2012) and that is also modulated by EE (Golabek et al., 2011; Pons-Espinal et al., 2013). Recently, we showed that combined treatment with EE and EGCG is more efficient than EE or EGCG alone to ameliorate age-associated cognitive impairment of older Ts65Dn mice (Catuara-Solarz et al., 2015), suggesting a synergistic mechanism. Furthermore, we demonstrated that combined treatment with cognitive training and EGCG is more efficient than cognitive training alone to promote cognitive enhancement as well as neurophysiological recovery in young adults with DS in a phase II clinical trial (de la Torre et al., 2016). Thus, here we explored the effects of combined EE-EGCG treatment on hippocampal cognitive, neuronal, and synaptic alterations in young adult Ts65Dn mice.

## Materials and Methods

### Animals

Ts65Dn (TS) and wild-type (WT) littermates were obtained through crossings of B6EiC3Sn a/A-Ts(17^16^)65Dn (Ts65Dn) females to B6C3F1/J males purchased from the Jackson Laboratory (Bar Harbor, ME; RRID:IMSR_JAX:001924). The mouse colony was bred in the Animal Facilities of the Barcelona Biomedical Research Park (PRBB, Barcelona, Spain). Mice were housed in standard or enriched conditions (see below) under a 12:12-h light:dark schedule (lights on at 8:00 a.m.) in controlled environmental conditions of humidity (60%) and temperature (22°C ± 2°C) with *ad libitum* access to food and water. Both the Ts65Dn and euploid mice were genotyped by quantitative PCR, in accordance with the Jackson Laboratory protocol.

Experiments were conducted using 1- to 2-month-old female mice. We used females because in EE conditions, Ts65Dn males show high levels of stress that could mask the effect of the treatment (Martínez-Cué et al., 2002). Although the estrus cycle may be slightly delayed in Ts65Dn mice, by the age of 2 months it is synchronized among all females (including Ts65Dn and euploid mice; Netzer et al., 2010). Thus, for the experiments conducted in this study, it is unlikely that variations in estrogen levels between mice could influence behavior, spine density, or E/I balance.

All animal procedures met the guidelines of European Community Directive 2010/63/EU and local guidelines (Real Decreto 53/2013) and were approved by the local ethics committee (Comité Ético de Experimentación Animal del PRBB; procedure numbers MDS-08-1060P2 and MDS-14-1611).

### EE and EGCG

Ts65Dn and WT 1- to 2-month-old female mice were assigned using a simple randomization to either control conditions or a combination of EE and green tea extracts containing 45% EGCG. Mice received the treatments for 30 days based on previous studies (De la Torre et al., 2014; Catuara-Solarz et al., 2015). In the control conditions, animals were reared in conventional Plexiglas cages (20 × 12 × 12-cm height) in groups of 2–3 animals. EE housing consisted of spacious (55 × 80 × 50-cm height) cages with toys, small houses, tunnels, and platforms of different shapes, sizes, colors, and textures. Wheels were not introduced in the cages to avoid the effect of physical exercise. The arrangement was changed every 2 days to keep novelty conditions. To stimulate social interactions, 6–8 mice were housed in each cage. Green tea extract containing 45% EGCG was administered in drinking water (EGCG dosage: 0.326 mg/ml, 0.65 mg per day; 30 mg/kg per day) by preparing fresh EGCG solution every 2 days from a green tea leaf extract (Mega Green Tea Extract, Decaffeinated, Life Extension, Fort Lauderdale, FL; EGCG content 326.25 mg per capsule).

### Morris water maze

The Morris water maze (MWM) was performed according to a previously described method (Catuara-Solarz et al., 2015). Briefly, mice were trained in a water maze (pool, 1.70-m diameter; platform, 12-cm diameter) during five learning sessions (four acquisition trials per session and one session per day). Twenty-four hours after the final acquisition session, mice underwent one probe/removal session (reference memory trial) in which the platform was removed, followed by one cued session. Starting the next day, three reversal sessions (four trials per session) were conducted in which the platform position was changed 180° to test cognitive flexibility as a measure of executive function. In every session, mice randomly entered the pool from four different positions and were allowed to search the platform for 60 s. The experimenter who performed all the MWM procedures was blind to mice genotype. Mice were video-tracked during the test, and their latency to reach the platform, total distance swum, time spent in periphery, and swimming speed were recorded using SMART software (Panlab, Barcelona, Spain, RRID:SCR_002852). Subsequently, data were computed with software developed by our lab (Jtracks; Arqué et al., 2008) to obtain other measurements to quantify the most efficient and direct trajectory from the location of mice to the platform, such as the Gallagher index (average distance from each mouse to the center of the platform), the Gallagher distance (accumulated distance from each mouse to the center of the platform), and the Whishaw index (percentage of path inside the optimal corridor connecting release site and goal; Whishaw and Jarrard, 1996). Mice that did not reach the platform in <30 s in the cue session were considered unsuitable for the test and were subtracted from the analysis. One overperforming mouse from the EE-EGCG–treated TS group was removed from the analysis. The estimate of the number of mice required (*n* = 10) was based on the expected difference between the experimental groups, deriving from previous data obtained in our laboratory (WT, *n* = 11; TS, *n* = 8; WT-EE-EGCG, *n* = 12; TS-EE-EGCG, *n* = 8).

### Novel object recognition test

The novel object recognition test was performed as an adaptation from the protocols described in Leger et al. (2013). The procedure was conducted in a V-maze apparatus (wall height 27 cm, arm length 30 cm, and arm width 6 cm). First, mice were subjected to a 5-min habituation session during which they were allowed to explore the maze without any objects. The next day, mice went through a 10-min familiarization session in which two identical objects were situated at the end of each arm attached to the wall and the floor with adhesive tape. After a 60-min intertrial interval, the recognition test session was conducted consisting of a 5-min trial in which one of the objects used at familiarization was substituted by a new object. Recognition of the new object was assessed by calculating the discrimination index (DI) by the following formula DI = (novel object exploration time/total exploration time) – (familiar object exploration time/total exploration time) × 100. Exploratory behavior was defined as the mouse directing its head or sniffing toward the object at a distance of ∼1–2 cm. The estimate of the sample size was based on previous data obtained in our laboratory (WT, *n* = 8; TS, *n* = 7; WT-EE-EGCG, *n* = 7; TS-EE-EGCG, *n* = 7).

### Golgi neuronal staining and dendritic spine imaging

To avoid confounding effects, these experiments were performed with mice that did not undergo behavioral assessment. Golgi staining was performed according to manufacturer instructions (SuperGolgi Kit, Bioenno Tech, Santa Ana, CA; cat. # 003010, RRID:AB_2620135). Mice were sacrificed with CO_2_ and perfused intracardially with 0.01 m PBS followed by chilled 4% paraformaldehyde (PFA). Brains were removed from the skull and hemispheres were immersed freshly into impregnation solution for 9 days. When impregnation was ready, tissue blocks were rinsed with distilled water and transferred into postimpregnation buffer for 4–5 days at room temperature (RT) in the dark. The solution was renewed after 1 day of immersion. After that, brains were cut with a vibratome (VT1000S; Leica Microsystems, Wetzlar, Germany) in sections of 150 μm and kept in collection and mounting buffer. Sections were mounted on adhesive microscope slides, and gentle pressure was applied with filter paper over the sections to enhance adhesion. Slides were washed in 0.01 m PBS/0.3%Triton X-100 for 20–30 min and placed in the staining solution for 20 min in a closed dark jar. After that, slides were moved to the poststaining buffer for 20 min in a dark area and washed in 0.01 m PBS/0.3% Triton X-100 for 15 min. Slides were dried in a closed jar at RT for 1 d. Finally, sections were dehydrated in 100% ethanol for 5–10 min and cleared in xylene for another 10 min. Slides were covered with coverslips using mounting medium and were kept at RT in a dark area.

Images were acquired from outer molecular layer secondary apical dendrites of granule neurons (located 40–90 μm from the neuronal soma) of the dentate gyrus (DG) and secondary apical dendrites from pyramidal neurons from the cornu ammonis 1 (CA1; located 50–100 μm from the neuronal soma). These hippocampal regions were selected because they play a critical role in the process and storage of spatial information (Tsien et al., 1996; Deng et al., 2010). We used an Olympus BX51 microscope with 100× objective and Neurolucida software (11.03.1, MBF Bioscience, Williston, VT; RRID:SCR_001775). Quantification of dendritic spine density was performed on 20-μm dendritic segment lengths using NIH ImageJ software version 1.46a and Multipoint plugin. The criterion to define dendritic spines was the identification of tridimensional protrusions emerging from the dendritic shaft that could be visualized across the *z*-planes. For this experiment, we used three brain slices per mouse of the dorsal hippocampus (bregma: 1.82–1.94, Paxinos and Franklin, 2012) of five to six mice per experimental group. From each brain slice, two to three dendrites from each hippocampal subregion were imaged (number of dendrites in DG: WT, 46; TS, 42; WT EE-EGCG, 48; TS EE-EGCG, 42; number of dendrites in CA1: WT, 38; TS, 32; WT EE-EGCG, 31; TS EE-EGCG, 39).

### Immunohistochemical labeling of excitatory and inhibitory synaptic vesicle proteins

Synaptic modifications due to genotype and treatment were addressed by performing immunohistochemical labeling for vesicular glutamate transporter 1 (VGLUT1) and vesicular GABA transporter (VGAT). Animals were exposed to CO_2_ and afterward perfused intracardially with 0.01 m PBS, pH 7.5, followed by 4% PFA. Brains were removed, kept at 4°C in 4% PFA overnight, and transferred to a solution of 30% sucrose in PBS for 2 days. Series of coronal sections (40 μm) were obtained using a vibratome (VT1000S; Leica Microsystems) and stored at –20°C in a cryoprotection solution (30% ethylene glycol, 30% glycerol, 40% PBS). Free-floating brain sections were permeabilized with 0.3% Triton X-100 in PBS for 30 min at RT and incubated for 20 min with Glycine (50 mm) in PBS/0.3% Triton X-100. Slices were washed for 15 min with PBS/0.3% Triton X-100 and blocked with 5% normal goat serum (NGS)/PBS/-0.3% Triton X-100 for 1 h at RT. Sections were incubated overnight at 4°C with the primary antibodies—mouse anti-VGLUT1 monoclonal antibody (1:150; cat. no. 135-511, Synaptic Systems, Goettingen, Germany; RRID: AB_887879) and guinea pig anti-VGAT polyclonal antibody (1:200; cytoplasmic domain; Synaptic Systems; cat. no. 131004, RRID:AB_887873)—in 0.1% Triton X-100/2.5% NGS in PBS. Slices were washed with PBS/0.3% Triton X-100 and incubated with secondary antibodies—Alexa Fluor 488 goat anti-mouse (cat. no. A11001, RRID:AB_2534069) and Alexa Fluor 555 anti–guinea pig (cat. no. A-21435, RRID:AB_2535856; 1:1000—in 0.1% Triton X-100/2.5% NGS in PBS for 2 h at RT, protected from light. Finally, sections were washed with PBS/0.3% Triton X-100, nuclei were stained with Hoechst (1:1000) in PBS for 10 min, and tissues were mounted on glass slides with Mowiol reagent.

Images were acquired from DG and CA1 hippocampal regions using a confocal microscope with a 63× objective (TCS SP5, Leica Microsystems) and LAS AF software. For each region, all pictures were captured with identical confocal settings for laser power, gain, and offset levels. Images were imported into ImageJ, converted into binary data, and thresholded to achieve maximum number of individual puncta without causing puncta fusion. The same threshold was applied to all the images to outline puncta number, size, and percentage of area occupied by puncta, using the “analyze particle” plugin. For this experiment, we used two brain slices of the dorsal hippocampus (bregma: 1.82–1.94, Paxinos and Franklin, 2012) of four mice per experimental group. From each brain slice, four to five images were acquired per hippocampal subregion.

## Statistical analysis

### Morris water maze

#### Single-variate analysis

Differences among experimental groups over time were tested using a single-variate analysis for selected learning-related parameters (latency to reach the platform, Gallagher index, and percentage of time spent in the periphery) using one-way repeated-measures ANOVA. To avoid the ceiling effect of mice unable to solve the task, the variable latency was considered right-censored when reaching the maximum allowed time (60 s; Vock et al., 2012) using a Tobit model implemented in the censReg package from R Foundation for Statistical Computing (RRID:SCR_001905), version 3.2.1 (Henningsen, 2011). Multiple comparisons for parametric models were used to address post hoc comparisons using the multtest R package and the glht function (Hand and Taylor, 1987; Dickhaus, 2012). To control the FDR caused by multiple post hoc comparisons, the Benjamini–Hochberg method was used (Benjamini and Hochberg, 1995).

#### Principal component analysis

We used principal component analysis (PCA) to identify the linear combinations of the original variables (latency to reach the platform, percentage of time spent in target quadrant, percentage of time spent in the periphery, Whishaw index, Gallagher index, distance traveled, and speed) that explain the maximum amount of experimental variability. More precisely, our application of the method aims primarily at two kinds of variability: variation among experimental groups for a given learning session and variation of a given group across learning sessions. For this, we implemented the same procedure as described in Catuara-Solarz et al. (2015). Briefly, a PCA for the acquisition sessions was performed on 20 observations of seven variables. Here, the observations correspond to the four experimental groups on the five learning sessions, and the variables are the experimental parameters described above. The resulting 20 × 7 data matrix contains the medians of the measured variables for each group during each session (the four trials per mouse of each learning session were averaged). As variables are measured in different units, they were scaled to unit variance to enable a combined analysis. The result of the described PCA approximates a decomposition of what is commonly called between-group variance. A third kind of variability coming from individuals within a group for a given session can also be quantified. For this, 195 supplementary points that correspond to the 39 individuals appearing five times each were projected. The R package FactoMineR was used (Lê et al., 2008). Separately, a similar PCA was done for the three reversal sessions.

Our analysis can be considered a discriminant analysis in the sense that the PCA is performed for groups and individuals are projected only after the PCA is performed. However, we use group medians instead of the commonly applied group means weighted by group sizes, leading to two differences: first, between-group variance is defined as variance between group medians; second, the overall barycenter no longer coincides with the group barycenter (our origin), and thus the total variance obtained by summing squared distances of all individuals from the origin as applied in Catuara-Solarz et al. (2015) overestimates the true variance by a small amount (i.e. by the squared distance of the barycenter from the origin). To comply with the original definition (Greenacre, 2010, chapter 11) of between-group variance when decomposing total variance, the overall barycenter instead of the origin can be used as the reference point, and weighted group means obtained from the supplementary points can be used to calculate the between-group variance instead. We found that the difference between the two approaches is on the order of a few percentage points only.

To validate the stability of the PCA, we used a jackknifing procedure: each individual is subtracted from the analysis, the resulting modified group median is used to perform a new PCA, and the angle between the new first principal component (PC1) and PC2 with respect to the original principal axes is computed. The procedure showed that both axes remain very stable, with PC1 attaining maximum angles around 1 degree, suggesting a minor influence of the small number of experimental groups on the outcome of the analysis (data not shown).

Density plots were obtained using the statdensity_2d function from the ggplot2 R package (Wickham, 2009; RRID:SCR_014601) with the parameters: *n* = 100, h = 4, and bins = 6. to assess statistical significance of group separation, we randomly reassigned individuals to experimental groups to perform a permutation test (Sham and Purcell, 2014) in which original numbers of individuals in each group were kept. For this, learning differences were evaluated using a *t* statistic involving PC1 pairwise group comparisons based on supplementary points. All pairwise comparisons were determined at each permutation. The number of randomized PCAs was 10,000. Finally, to evaluate the change in within-group variances before and after learning, we averaged squared distances of a group’s supplementary points from their barycenter using coordinates from all seven principal axes.

### Novel object recognition

Differences in the discrimination index among experimental groups were tested using one-way ANOVA. Tukey multiple comparisons for parametric models were used to address post hoc comparisons using the multtest R package and the glht function (Hand and Taylor, 1987; Dickhaus, 2012). To control the FDR caused by multiple post hoc comparisons, the Benjamini–Hochberg method was used (Benjamini and Hochberg, 1995).

### Dendritic spine density and excitatory (VGLUT1) and inhibitory (VGAT) synaptic puncta

For analysis of the differences among the experimental groups in dendritic spine densities and number, size, and percentage of area occupied by synaptic puncta of VGLUT1 and VGAT, we used linear mixed models, which included experimental group as a factor and mouse as a random effect to account for the repeated measures per mouse. The *F* test was used to evaluate the global hypothesis that there was no association between the response variables and the groups. Whenever this hypothesis was rejected, post hoc tests for the following contrasts of interest were applied: WT versus TS; TS versus TS EE-EGCG, and WT versus WT-EE-EGCG. The analyses were performed using R packages nlme (Pinheiro et al., 2016) and multcomp (Hothorn et al., 2008) for the fit of the linear mixed models and the multiple tests, respectively. Statistical significance was set at 0.05. The significance levels for the three contrasts of interest were adjusted to guarantee a family-wise error rate of 0.05.

## Results

### Effects of EE-EGCG treatment on corticohippocampal-dependent learning and memory impairment in Ts65Dn mice

To evaluate the effect of EE-EGCG treatment, we compared the behavioral performance of WT and Ts65Dn mice treated with EE-EGCG with their untreated counterparts in the MWM. During the acquisition sessions, there were statistical differences among all groups in escape latency, distance to the target [as shown by both the Gallagher index (mean distance to the platform) and the Gallagher (accumulated) distance to the platform], and thigmotactic behavior, (percentage of time spent close to the periphery) the percentage of time spent close to the periphery of the pool (Table 1).

**Table 1.** Single-variate MWM multiple post hoc comparisons with Benjamini–Hochberg correction.

								95% confidence interval		
Figure	Variable	Phase	Contrast	Data structure	Type of test	Estimated mean difference	SE	Lower	Higher	*p*-value
Fig. 1B	Latency	ACQ	Overall effect	Continuous variable	Tobit model	χ^2^_(3)_ = 42.24				<0.001
Fig. 1B	Latency	ACQ	TS_WT	Continuous variable	Tobit model	51.262	11.28	29.146	73.377	<0.001
Fig. 1B	Latency	ACQ	TS EE-EGCG_TS	Continuous variable	Tobit model	–23.839	12.98	–49.280	1.602	0.028
Fig. 1B	Latency	ACQ	TS EE-EGCG_WT	Continuous variable	Tobit model	27.423	11.40	5.066	49.780	0.005
Fig. 1C	Gallagher distance	ACQ	Overall effect	Continuous variable	ANOVA repeated-measures *F*-test	*F* = 13.636				<0.001
Fig. 1C	Gallagher distance	ACQ	TS _WT	Continuous variable	ANOVA repeated-measures *F*-test	1903.3	397.6	1124	2682.6	<0.001
Fig. 1C	Gallagher distance	ACQ	TS EE-EGCG_TS	Continuous variable	ANOVA repeated-measures *F*-test	–896.3	427.9	–1734.9	–57.6	0.043
Not shown	Gallagher index	ACQ	Overall effect	Continuous variable	ANOVA repeated-measures *F*-test	*F* = 10.226				<0.001
Not shown	Gallagher index	ACQ	TS _WT	Continuous variable	ANOVA repeated-measures *F*-test	17.217	3.981	9.41424	25.01976	<0.001
Not shown	Gallagher index	ACQ	TS EE-EGCG _TS	Continuous variable	ANOVA repeated-measures *F*-test	–8.903	4.284	–17.2996	–0.50636	0.045
Fig. 1D	Thigmotaxis	ACQ	Overall effect	Continuous variable	ANOVA repeated-measures *F*-test	*F* = 9.22				<0.001
Fig. 1D	Thigmotaxis	ACQ	TS_WT	Continuous variable	ANOVA repeated-measures *F*-test	21.172	5.629	10.13916	32.20484	<0.001
Fig. 1D	Thigmotaxis	ACQ	TS EE-EGCG _TS	Continuous variable	ANOVA repeated-measures *F*-test	–11.374	6.057	–23.2457	0.49772	0.09
Not shown	Speed	ACQ	Overall effect	Continuous variable	ANOVA repeated-measures *F*-test	*F* = 4.883				0.006
Not shown	Speed	ACQ	TS_WT	Continuous variable	ANOVA repeated-measures *F*-test	–4.547	1.354	–7.20084	–1.89316	0.004
Not shown	Speed	ACQ	TS_TS EE-EGCG	Continuous variable	ANOVA repeated-measures *F*-test	1.725	1.457	–1.13072	4.58072	0.28
Not shown	Speed	ACQ	WT EE-EGCG _WT	Continuous variable	ANOVA repeated-measures *F*-test	–0.485	1.216	–2.86836	1.89836	0.69
Fig. 1E	Latency first entry	REM	TS_WT	Continuous variable	One-way ANOVA	25.284	7.295	10.9858	39.5822	0.004
Fig. 1E	Latency first entry	REM	TS EE-EGCG _TS	Continuous variable	One-way ANOVA	–16.075	7.850	–31.461	–0.689	0.096
Fig. 1F	Latency first entry	REM	Overall effect	Continuous variable	One-way ANOVA	*F* = 6.159				0.002
Fig. 1F	Gallagher index	REM	TS_WT	Continuous variable	One-way ANOVA	17.991	7.041	4.19064	31.79136	0.03
Fig. 1F	Gallagher distance	REM	TS_WT	Continuous variable	One-way ANOVA	1272.4	455.5	–437.28	2165.18	0.025
Fig. 1F	Gallagher index	REM	TS EE-EGCG _TS	Continuous variable	One-way ANOVA	–11.944	7.577	–26.7949	2.90692	0.14
Fig. 1G	Latency	REV	Overall effect	Continuous variable	Tobit model	χ^2^_(3)_ = 26.59				<0.001
Fig. 1G	Latency	REV	TS_WT	Continuous variable	Tobit model	35.093	9.16	17.124	53.063	<0.001
Fig. 1G	Latency	REV	TS EE-EGCG _TS	Continuous variable	Tobit model	–14.865	9.28	–33.073	3.342	0.060
Fig. 1H	Gallagher distance	REV	Overall effect	Continuous variable	ANOVA repeated-measures *F*-test	*F* = 7.694				<0.001
Fig. 1H	Gallagher distance	REV	TS_WT	Continuous variable	ANOVA repeated-measures *F*-test	2114.3	412	1306.78	2921.82	<0.001
Fig. 1H	Gallagher distance	REV	TS EE-EGCG _TS	Continuous variable	ANOVA repeated-measures *F*-test	–869	443.3	–1737.87	–0.132	0.059
Not shown	Gallagher index	REV	Overall effect	Continuous variable	ANOVA repeated-measures *F*-test	*F* = 11.714				<0.001
Not shown	Gallagher index	REV	TS_WT	Continuous variable	ANOVA repeated-measures *F*-test	20.8045	4.3102	12.35651	29.25249	<0.001
Not shown	Gallagher index	REV	TS EE-EGCG _TS	Continuous variable	ANOVA repeated-measures *F*-test	–6.3488	4.638	–15.4393	2.74168	0.2
Fig. 1H	Thigmotaxis	REV	Overall effect	Continuous variable	ANOVA repeated-measures *F*-test	*F* = 10.105				<0.001
Fig. 1H	Thigmotaxis	REV	TS_WT	Continuous variable	ANOVA repeated-measures *F*-test	21.393	6.268	9.10772	33.67828	0.001
Fig. 1H	Thigmotaxis	REV	TS EE-EGCG _TS	Continuous variable	ANOVA repeated-measures *F*-test	–9.867	6.745	–23.0872	3.3532	0.14
Not shown	Speed	REV	Overall effect	Continuous variable	ANOVA repeated-measures *F*-test	*F* = 2.607				0.067

**Figure 1.**
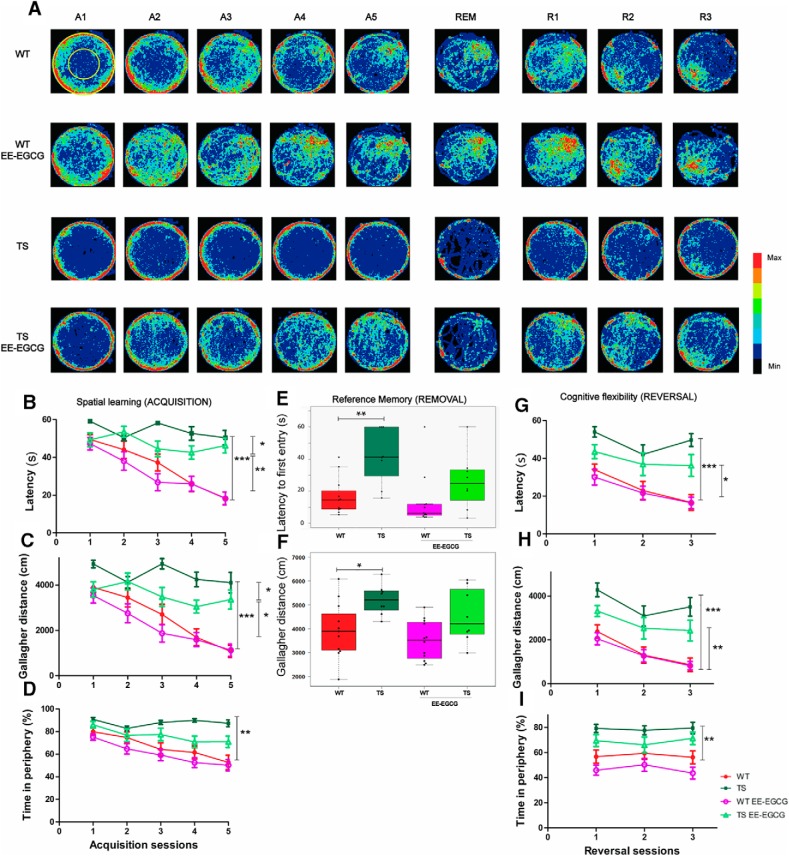
**EE–EGCG treatment effects on young Ts65Dn mice deficits in spatial learning, reference memory and cognitive flexibility.** ***A***, Heat map representing the accumulated swimming trajectories of mice from the different experimental groups across acquisition, removal, and reversal sessions in the MWM. Periphery and center zones are depicted at upper left. Color code is represented on the right, with red corresponding to the most visited zones and black to less visited or nonvisited zones. Learning curves are represented in latency (s) to reach the escape platform (***B***), Gallagher distance (accumulated distance to the goal in cm; ***C***), and thigmotaxis (percentage of time spent on the periphery; ***D***) during the acquisition sessions. ***E***,***F***, Boxplots of the distribution of latency to the first entry to the platform area and the Gallagher distance of the four experimental groups in the removal session. Dots indicate the values of each individual mouse. Reversal learning curves are represented in latency (s; ***G***), Gallagher distance (***H***), and thigmotaxis (***I***). A1-5, acquisition sessions 1–5; R1-3, reversal sessions 1–3 with four trials per day; REM, removal session. Data in ***B***, ***C***, ***D***, ***F***, ***G***, and ***H*** are mean ± SEM. Data in ***B*** and ***F*** were analyzed by a censored model, which considered 60 s as the maximum trial duration. Data in ***C***, ***D***, ***G***, and ***H*** were analyzed with ANOVA repeated measures, and data in ***E*** were analyzed by one-way ANOVA. In all cases, Tukey multiple post hoc comparisons corrected with Benjamini–Hochberg were used. Even if all groups were considered for multiple comparisons, the figure reports only statistically significant differences of the following relevant contrasts of interest: WT versus TS; TS versus TS EE-EGCG; WT versus TS EE-EGCG. *p < 0.05, **p < 0.01, ***p < 0.001.

We detected learning defects in Ts65Dn mice compared with WT (Fig. 1*A*) as shown by the higher escape latency across sessions (Fig. 1*B*; Table 1), increased Gallagher distance and index (Fig. 1*A*, *C*; Table 1), and typical increase in thigmotactic behavior (Fig. 1*A*, *D*; Table 1).

EE-EGCG–treated Ts65Dn mice showed improved learning performance during the acquisition sessions (Fig. 1*A*). In comparison to untreated Ts65Dn mice, EE-EGCG–treated TS mice presented significantly reduced escape latency (Fig. 1*B*; Table 1) and Gallagher distance and index (Fig. 1*A*, *C*; Table 1) but no statistical differences in thigmotactic behavior (Fig. 1*A*, *D*; Table 1). Conversely, EE-EGCG–treated WT mice did not show differences compared with untreated WT mice.

There were differences in swimming speed among the groups; specifically, Ts65Dn mice had lower swimming speeds than WT mice (data not shown; Table 1). However, EE-EGCG did not promote significant changes in swimming speed in Ts65Dn or WT with respect to the untreated condition (data not shown; Table 1). This suggests that the learning differences in EE-EGCG–treated Ts65Dn mice are not mediated by changes in swimming speed.

To assess reference memory, a probe trial (removal session) was performed 24 h after the final acquisition day. In this session, there were no differences among groups in the percentage of time spent in the target quadrant, probably because of the high variability of the data (data not shown). However, the latency to the first entry to the platform area and the Gallagher distance, which is a more precise performance measure (Maei et al., 2009), showed significant differences among experimental groups (Fig. 1*A*, *E*, *F*; Table 1). Post hoc analysis demonstrated higher values of Ts65Dn mice in the Gallagher distance and the latency to the first entry compared with WT mice (Fig. 1*A*, *E*, *F*; Table 1), indicating poorer reference memory. The mean difference in these parameters between EE-EGCG–treated and untreated Ts65Dn mice was fairly large; however, it did not reach statistical significance, possibly because of high variability of the data (Fig. 1*A*, *E*, *F*; Table 1).

In the reversal sessions, we detected statistical differences among the experimental groups in escape latency, Gallagher distance and index, and thigmotaxis (Table 1). Whereas untreated WT mice efficiently shifted their search to the new platform position (Fig. 1*A*), Ts65Dn mice had increased escape latency (Fig. 1*G*; Table 1), increased Gallagher distance and index (Fig. 1*A*, *H*; Table 1), and increased thigmotaxis across the three reversal learning sessions (Fig. 1*A*, *I*; Table 1), compared with WT mice. During reversal, there was no significant reduction in thigmotaxis, suggesting that this variable was not associated with reversal learning.

EE-EGCG–treated Ts65Dn mice showed a fairly large (although not statistically significant) reduction in the latency to reach the new platform position compared with untreated Ts65Dn mice (Fig. 1*A*, *G*; Table 1). There were no significant differences between EE-EGCG–treated and untreated Ts65Dn mice in Gallagher distance or index (Fig. 1*A*, *H*; Table 1) or thigmotaxis (Fig. 1*A*, *I*; Table 1). EE-EGCG–treated and untreated WT mice showed no significant differences in the latency to reach the new platform position, the Gallagher distance or index, or thigmotaxis. During the reversal sessions, there were no statistical differences in swimming speed among the experimental groups (not shown; Table 1).

### Multidimensional analysis of learning using PCA

**Figure 2.**
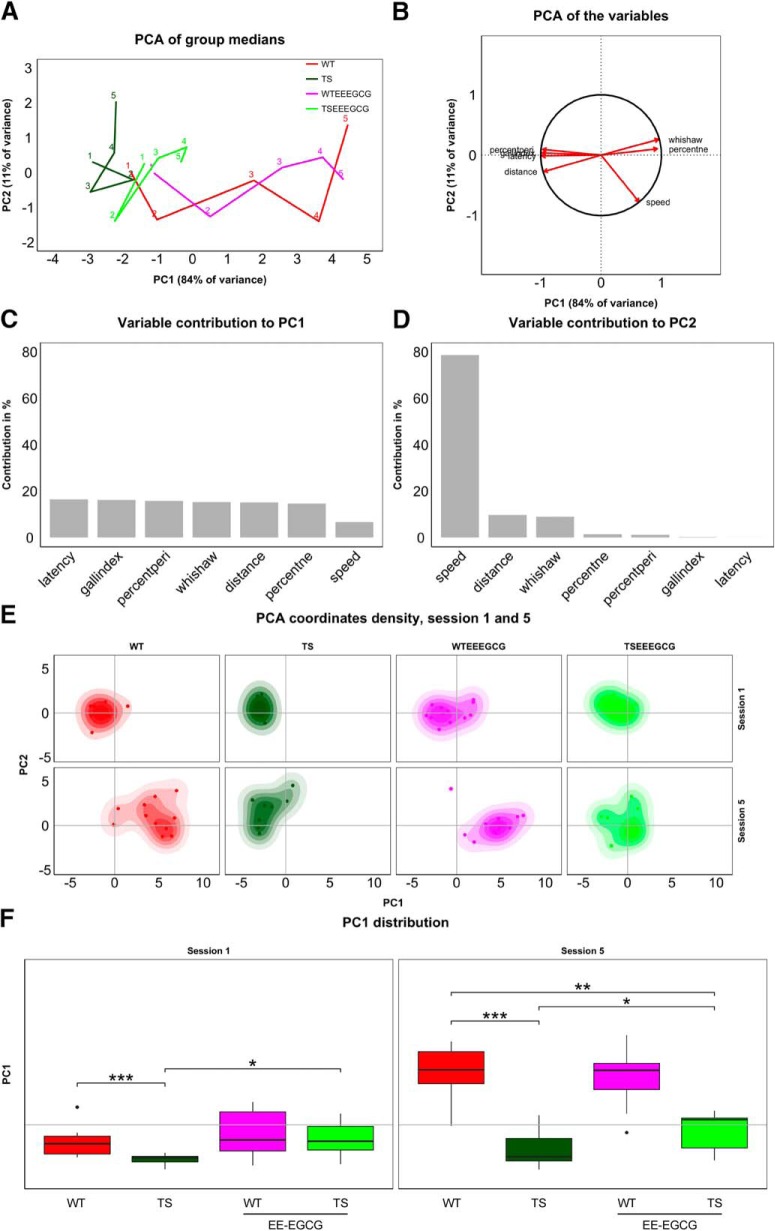
Supervised PCA of the experimental groups during the acquisition sessions revealed that EE-EGCG treatment improves global learning in Ts65Dn mice. ***A***, Trajectories connect group performance (medians) along the five learning sessions (labeled with respective numbers) in the space defined by PC1 and PC2, which consist of linear combinations of the original variables. ***B***, Variable directionality in the PCA space. Arrows represent the direction with respect to PC1 and PC2. Variables reaching the unit circle belong to variables that are well represented by the two principal components. Contribution of variables to PC1 (***C***) and PC2 (***D***) in percent. PC1 receives a similar contribution from all classic variables used to assess learning and can thus be understood as a composite learning variable. ***E***, Boxplots of PC1 distribution for each experimental group on sessions 1 and 5 of the acquisition phase. Box plot horizontal lines, group median; box edges, 25th and 75th percentiles; whiskers, minimum and maximum values to a maximum of 1.5 times the interquartile distance from the box. More extreme values are individually plotted. Only relevant comparisons are reported in the figure for the sake of clarity (WT versus TS; TS versus TS EE-EGCG; WT versus TS EE-EGCG), even if all groups were considered for the permutation test. *p < 0.05, **p < 0.01,***p < 0.001. The benefits of the EE-EGCG treatment on Ts65Dn learning explain the displacement of this group toward more positive values along the PC1.

PCA enabled placement of the experimental groups in a low-dimensional coordinate system built from variables taken during the MWM experiment. A group’s progression along the acquisition sessions became apparent in its resulting 5-day trajectory (Fig. 2*A*). We obtained a PC1 that explained 84% of the between-group variance and was mainly composed of learning-related variables (i.e., escape latency, Gallagher index, percentage of time spent in periphery, Whishaw index, distance traveled, percentage of time spent in target quadrant; Figs. 2*B*, *C*). Swimming speed also contributed to PC1, but to a lesser extent. Efficient learning behaviors (short distances to target, low escape latencies, high percentages of time in the target quadrant, etc.) correspond to large values of PC1 (Fig. 2*B*), and thus PC1 can be interpreted as a quantification of learning. In contrast, PC2 explained 11% of between-group variance and was mainly composed of swimming speed (Figs. 2*B*, *D*). This component of speed is unrelated to learning, since PC2 is orthogonal to the learning-related PC1. It thus seems to reflect motor performance rather than determination to reach the target quickly. Swimming speed is thus decomposed in a learning-dependent component (PC1) and a learning-independent component (PC2). Learning-related variables contributed to a much lesser extent to PC2.

Because a group trajectory represents a group’s overall learning through its progression along PC1, the trajectory representation allows for effective comparisons between group performances. Untreated Ts65Dn mice showed a trajectory reaching a maximum value of PC1 that corresponds to initial PC1 values of the untreated WT trajectory, revealing poor learning. On the other hand, the EE-EGCG–treated Ts65Dn trajectory attained more advanced maximum values of PC1.

Additionally, we determined individual variation within the groups by mapping the position of each individual on each acquisition day on the PCA plot. As shown in the density plots of sessions 1 and 5 in Fig. 2*E*, there is substantial individual variation across learning sessions in all the experimental groups. In fact, overall group differences explain less than half of the total variance. Within-group variance, however, differs between experimental groups. Whereas WT mice generally show higher variability than Ts65Dn mice, the treatment roughly doubles variance for both genotypes in the first learning session. Interestingly, learning increases variability in all groups. This effect is stronger for untreated groups, so treated and untreated groups show similar variability in the final learning session. We summarize within-group variances of the first and last acquisition sessions in Table 2.

**Table 2.** Multivariate within-group variances (sum over squared distances from group barycenter divided by group size, scaled by number of variables) before and after acquisition, and reversal sessions.

Figure	Session	WT	WT-EE-EGCG	TS	TS-EE-EGCG
Fig. 2E	ACQ1	0.42	0.89	0.24	0.53
Fig. 2E	ACQ5	1.62	1.55	0.87	0.95
Fig. 3E	REV1	1.52	1.12	0.56	1.22
Fig. 3E	REV3	2.68	1.84	1.42	2.26

To assess the statistical significance of differences in learning, we performed a permutation test involving a *t* statistic based on PC1. Untreated Ts65Dn mice had significantly lower PC1 values in comparison to WT mice in the first learning session (Fig. 2*F*; Table 3). EE-EGCG–treated Ts65Dn mice had higher PC1 than untreated Ts65Dn mice (Fig. 2*F*; Table 3); at this stage, the difference could be associated with procedural learning and was not significantly different from untreated WT (Fig. 2*F*; Table 3). At the end of the learning period (session 5), untreated Ts65Dn mice still had significantly lower PC1 values in comparison to WT mice (Fig. 2*F*; Table 3). EE-EGCG–treated Ts65Dn mice exhibited higher PC1 than untreated Ts65Dn mice, although they showed significantly lower PC1 values than untreated WT mice (Fig. 2*F*; Table 3). On the other hand, EE-EGCG treatment did not significantly change learning outcomes of WT mice in either the first or the last session.

**Table 3.** Permutation-test results of learning-related composite measure PC1.

Figure	Variable	Phase	Contrast	Pseudo-*t*	*p*-value
Fig. 2E	PC1	ACQ1	TS_WT	3.67	<0.001
Fig. 2E	PC1	ACQ1	TS_WT EE-EGCG	3.57	<0.001
Fig. 2E	PC1	ACQ1	TS_TS EE-EGCG	3.05	0.004
Fig. 2E	PC1	ACQ1	TS EE-EGCG_WT	0.28	0.39
Fig. 2E	PC1	ACQ1	TS EE-EGCG_WT EE-EGCG	0.54	0.71
Fig. 2E	PC1	ACQ1	WT_WT EE-EGCG	0.8	0.89
Fig. 2E	PC1	ACQ5	TS_WT	6.81	<0.001
Fig. 2E	PC1	ACQ5	TS_WT EE-EGCG	6.72	<0.001
Fig. 2E	PC1	ACQ5	TS_TS EE-EGCG	1.85	0.045
Fig. 2E	PC1	ACQ5	TS EE-EGCG_WT	5.2	9.99
Fig. 2E	PC1	ACQ5	TS EE-EGCG_WT EE-EGCG	5.06	<0.001
Fig. 2E	PC1	ACQ5	WT_WT EE-EGCG	0.27	0.39
Fig. 3E	PC1	REV1	TS_WT	4.60	<0.001
Fig. 3E	PC1	REV1	TS_WT EE-EGCG	5.60	<0.001
Fig. 3E	PC1	REV1	TS_TS EE-EGCG	2.59	0.01
Fig. 3E	PC1	REV1	TS EE-EGCG_WT	2.44	0.01
Fig. 3E	PC1	REV1	TS EE-EGCG_WT EE-EGCG	3.07	0.005
Fig. 3E	PC1	REV1	WT_WT EE-EGCG	0.23	0.58
Fig. 3E	PC1	REV3	TS_WT	5.17	<0.001
Fig. 3E	PC1	REV3	TS_WT EE-EGCG	6.19	<0.001
Fig. 3E	PC1	REV3	TS_TS EE-EGCG	2.32	0.02
Fig. 3E	PC1	REV3	TS EE-EGCG_WT	2.66	0.01
Fig. 3E	PC1	REV3	TS EE-EGCG_WT EE-EGCG	3.21	0.004
Fig. 3E	PC1	REV3	WT_WT EE-EGCG	0.21	0.58

**Figure 3.**
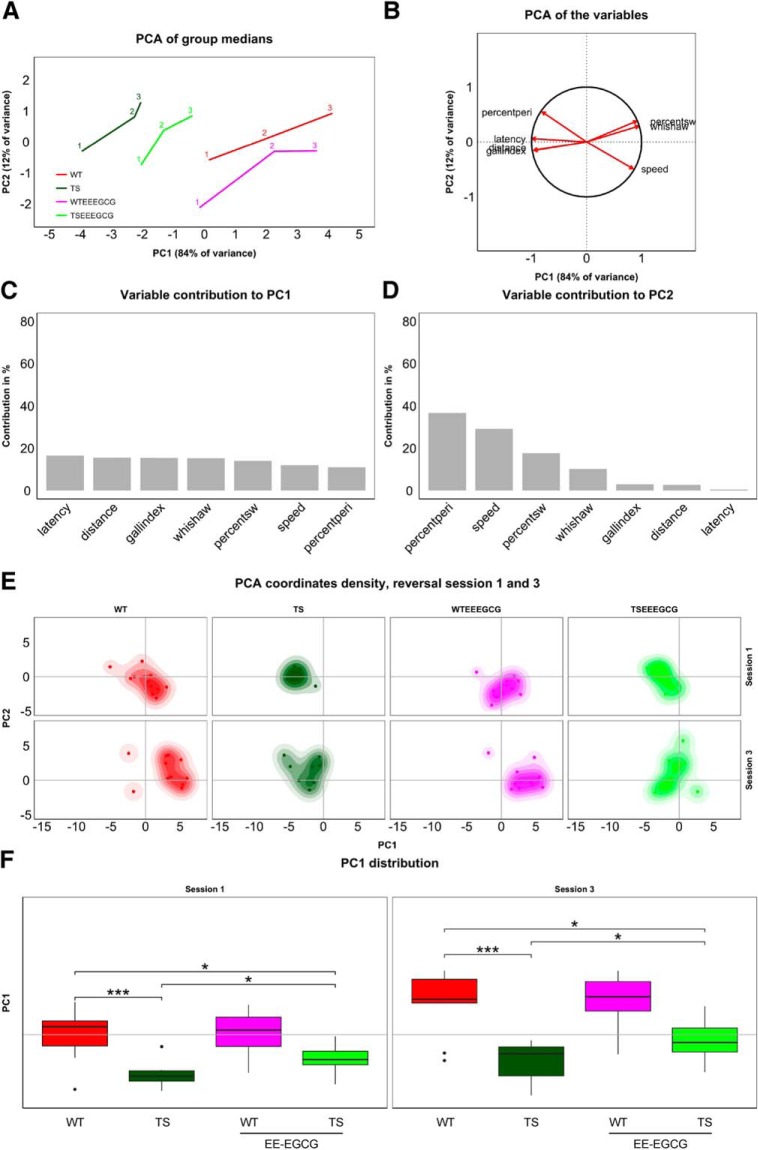
Supervised PCA of the experimental groups during the reversal sessions revealed poorer cognitive flexibility of untreated Ts65Dn mice. ***A***, Trajectories of medians along the reversal session (accordingly labeled) on the space formed by PC1 and PC2. ***B***, Variable directions on the PCA space defined by PC1 and PC2. Variables reaching the unit circle belong to variables that are well represented by the two principal components. Bar plots represent the contribution of variables to PC1 (***C***) and PC2 (***D***) in percent. ***E***, Box plots of PC1 distribution for each experimental group on sessions 1 and 3 of the reversal phase. Box plot horizontal lines, group median; box edges, 25th and 75th percentiles; whiskers, minimum and maximum values to a maximum of 1.5 times the interquartile distance from the box. More extreme values are individually plotted. Only relevant comparisons are reported in the figure for the sake of clarity (WT versus TS; TS versus TS EE-EGCG; WT versus TS EE-EGCG), even if all groups were considered for the permutation test. *p < 0.05, **p < 0.01,***p < 0.001. The shift of mouse groups toward positive values of PC1 represents the increased cognitive flexibility of the groups, with EE-EGCG–treated Ts65Dn mice attaining higher values than their untreated counterparts.

Similarly, group trajectories comprising three time points each were obtained for the reversal sessions (Fig. 3*A*). Here, PC1 explained 84% of the between-group variance and, as in the acquisition sessions, was dominated by learning-related variables (Fig. 3*B*, *C*). PC2, which explained 12% of the between-group variance, showed again a strong contribution of swimming speed. The main contribution here, however, turned out to be from thigmotaxis (Fig. 3*B*, *D*). Interestingly, these two variables fall on a line separating the groups along an efficiency gradient from strong thigmotaxis and low speed to no thigmotaxis and high speed in the order TS, TS-EE-EGCG, WT, and WT-EE-EGCG (Fig. 3*A*, *B*). Again, there was an increased within-group variability associated with the learning process in all groups (Fig. 3*E*; Table 2).

According to the permutation tests, untreated Ts65Dn mice showed significantly lower PC1 than untreated WT mice (Fig. 3*F*; Table 2). EE-EGCG–treated Ts65Dn mice had significantly higher PC1 than untreated Ts65Dn mice, although they still showed significantly lower PC1 than WT mice (Fig. 3*F*; Table 2). In WT mice, EE-EGCG treatment did not modify cognitive flexibility outcomes in the first or the last session.

### Effects of EE-EGCG treatment on recognition deficits in Ts65Dn mice

To assess the impact of the treatment on a less stressful learning task, we conducted a novel object recognition test. The performance of this test depends on the functionality of the entorhinal and perirhinal cortices and the hippocampus (Brown and Aggleton, 2001).

**Figure 4.**
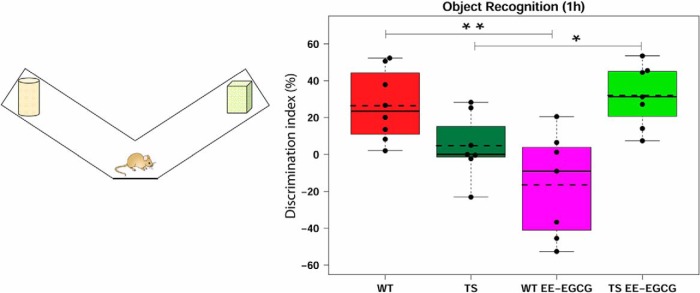
Effect of treatment on DI in the novel object recognition test. Left, diagram of the apparatus used for the novel object recognition test. Right, boxplots of the distribution of the DI (%) among the experimental groups. Dots, DI measure from each individual mouse; dashed lines, group means; continuous lines, group medians. Data were analyzed using one-way ANOVA and Tukey multiple post hoc comparisons corrected with Benjamini–Hochberg. Even if all groups were considered for multiple comparisons, the figure reports only statistically significant differences of the following relevant contrasts of interest: WT versus TS; TS versus TS EE-EGCG; WT versus TS EE-EGCG. *p < 0.05, **p < 0.01. Ts65Dn mice show a trend toward a reduction in DI (*p* = 0.08). EE-EGCG treatment improves DI score in Ts65Dn mice but worsens performance in WT mice.

In this test, Ts65Dn mice showed no deficit in their DI in comparison to WT mice, although a slight tendency to impairment was detected (*p* = 0.08; Fig. 4; Table 4). EE-EGCG–treated Ts65Dn mice had an increase in their DI with respect to their untreated counterparts (Fig. 4; Table 4) and scored at similar levels to WT mice (Fig. 4; Table 4). Conversely, EE-EGCG–treated WT mice showed a poorer performance than untreated WT mice (Fig. 4; Table 4).

**Table 4.** Novel object recognition test (discrimination index).

					95% confidence interval		
Figure	Contrast	Data structure	Type of test	Estimated mean difference	Lower	Higher	*p*-value
Fig. 4	TS _WT	Continuous variable	ANOVA	–21.728	–42.94	–0.511	0.083
Fig. 4	TS EE-EGCG_TS	Continuous variable	ANOVA	27.224	5.311	49.136	0.044
Fig. 4	TS EE-EGCG _WT	Continuous variable	ANOVA	5.496	–15.721	26.713	0.616
Fig. 4	WT EE-EGCG_WT	Continuous variable	ANOVA	–42.928	–64.145	–21.711	0.001

### Effects of EE-EGCG treatment on dendritic spine density in Ts65Dn hippocampus

**Figure 5.**
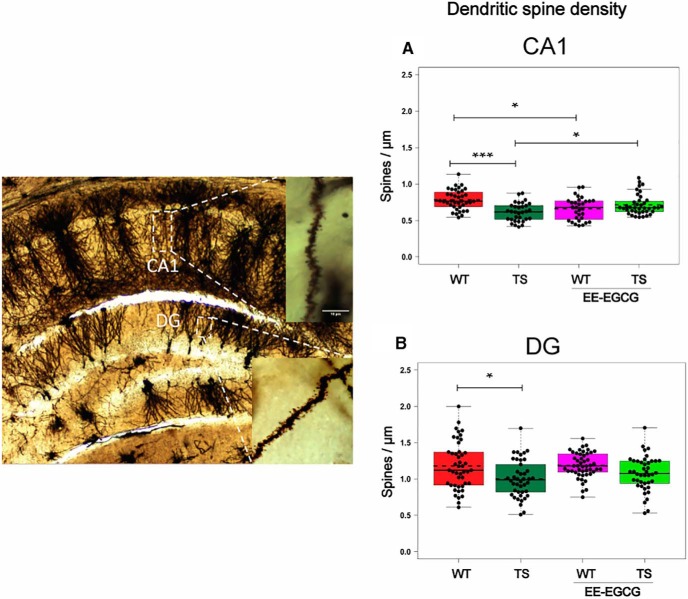
Ts65Dn mice show a reduction in dendritic spine density in DG and CA1, and EE-EGCG treatment ameliorates this deficit in CA1. Left, dorsal hippocampal region of a Golgi preparation illustrating dendrites from CA1 and DG subregions; scale bar represents 10 μm. ***A***,***B***, Boxplots of the distribution of dendritic spine density (spines per micrometer) in DG and CA1 among the experimental groups. Dots, repeated values from individual mice (two to three dendrites per slice, three dorsal hippocampal slices per brain, five to six mice per experimental group); dashed lines, group means; continuous lines, group medians. Data were analyzed with a linear mixed model, which included experimental group as a factor and mouse as a random effect. *F* test was used to test the global hypothesis. Post hoc tests were applied for the following contrasts of interest: WT versus TS; TS versus TS EE-EGCG; WT versus WT-EE-EGCG. *p < 0.05, ***p < 0.001. Ts65Dn mice show a significant reduction in spine density in both DG and CA1. EE-EGCG treatment increases dendritic spine density in Ts65Dn CA1 and decreases this parameter in WT.

Ts65Dn mice showed a significant reduction of dendritic spine density in the CA1 (Fig. 5*A*; Table 5) and DG (Fig. 5*B*; Table 5) hippocampal subregions. EE-EGCG–treated Ts65Dn mice did not show statistically significant differences in DG dendritic spine density compared with untreated Ts65Dn or WT mice (Fig. 5*B*). Conversely, EE-EGCG–treated Ts65Dn mice had increased dendritic spine density in CA1 in comparison to untreated Ts65Dn mice (Fig. 5*A*; Table 5), and EE-EGCG–treated WT mice showed reduced CA1 dendritic spine density in comparison to untreated WT mice (Fig. 5*A*; Table 5).

**Table 5.** Dendritic spine density.

						95% confidence interval		
Figure	Region	Contrast	Data structure	Type of test	Estimated mean difference	Lower	Higher	*p*-value
Fig. 5A	DG	TS _WT	Continuous variable	Mixed-model *F*-test	–0.192	–0.383	–0.002	0.048
Fig. 5B	CA1	TS _WT	Continuous variable	Mixed-model *F*-test	–0.175	–0.282	–0.069	<0.001
Fig. 5B	CA1	TS EE-EGCG_TS	Continuous variable	Mixed-model *F*-test	0.105	0.001	0.209	0.047
Fig. 5B	CA1	WT EE-EGCG_WT	Continuous variable	Mixed-model *F*-test	–0.129	–0.234	–0.025	0.01

### Effects of EE-EGCG treatment on hippocampal excitatory and inhibitory synaptic puncta in Ts65Dn mice

**Figure 6.**
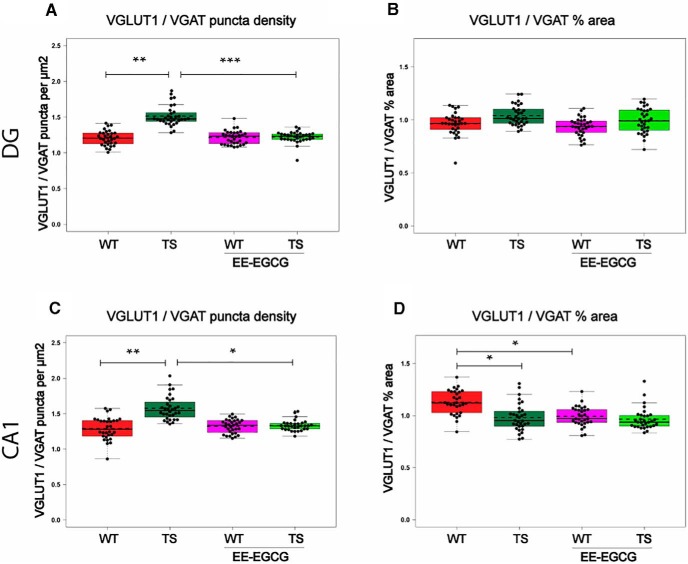
EE-EGCG effects on VGLUT1/VGAT puncta in DG and CA1. Box plots of the distribution of VGLUT1/VGAT ratios of different puncta density and percentage of area among the experimental groups at DG and CA1. ***A***, ***C***, VGLUT1/VGAT ratio of puncta density (puncta per square micrometer). ***B***, ***D***, VGLUT1/VGAT ratio of percentage of area occupied. Dots, repeated values from individual mice; dashed lines, group means; continuous lines, group medians. Data were analyzed with a linear mixed model, which included experimental group as a factor and mouse as a random effect. *F* test was used to test the global hypothesis. Post hoc tests were applied for the following contrasts of interest: WT versus TS; TS versus TS EE-EGCG; WT versus WT-EE-EGCG. *p < 0.05, **p < 0.01, ***p < 0.001.

In DG, Ts65Dn mice showed increased VGLUT1 puncta density (data not shown; Table 6) of reduced size (data not shown; Table 6), and no differences in the number or size of VGAT puncta compared with WT mice. This resulted in an increased VGLUT1/VGAT density ratio (Fig. 6*A*; Table 6). Because the increase in the number of VGLUT1 puncta was compensated by a reduction in size, Ts65Dn mice showed no difference in the VGLUT1/VGAT percentage of area occupied compared with WT mice (Fig. 6*B*; Table 6).

**Table 6.** VGLUT1 and VGAT synaptic puncta.

							95% confidence interval		
Figure	Variable	Region	Contrast	Data structure	Type of test	Estimated mean difference	Lower	Higher	*p*-value
Not shown	VGLUT1 puncta density	DG	TS _WT	Continuous variable	Mixed-model *F*-test	0.077	0.018	0.136	0.006
Not shown	VGLUT1 puncta size	DG	TS _WT	Continuous variable	Mixed-model *F*-test	–0.06	–0.098	–0.022	0.001
Fig. 6A	VGLUT1/VGAT puncta density	DG	TS _WT	Continuous variable	Mixed-model *F*-test	0.308	0.151	0.465	<0.001
Fig. 6B	VGLUT1/VGAT % of area	DG	TS _WT	Continuous variable	Mixed-model *F*-test	0.077	–0.03	0.183	0.222
Not shown	VGLUT1puncta density	DG	TS EE-EGCG_TS	Continuous variable	Mixed-model *F*-test	–0.073	–0.132	–0.014	0.01
Not shown	VGLUT1 puncta size	DG	TS EE-EGCG_TS	Continuous variable	Mixed-model *F*-test	0.06	0.022	0.098	0.001
Fig. 6A	VGLUT1/VGAT puncta density	DG	TS EE-EGCG_TS	Continuous variable	Mixed-model *F*-test	–0.294	–0.452	–0.137	<0.001
Not shown	VGLUT1 puncta density	CA1	TS _WT	Continuous variable	Mixed-model *F*-test	0.071	0.008	0.134	0.022
Not shown	VGLUT1 puncta size	CA1	TS _WT	Continuous variable	Mixed-model *F*-test	–0.093	–0.145	–0.041	0.043
Not shown	VGAT puncta size	CA1	TS _WT	Continuous variable	Mixed-model *F*-test	0.054	0.001	0.106	0.043
Fig. 6C	VGLUT1/VGAT puncta density	CA1	TS _WT	Continuous variable	Mixed-model *F*-test	0.295	0.098	0.493	0.001
Fig. 6D	VGLUT1/VGAT % of area	CA1	TS _WT	Continuous variable	Mixed-model *F*-test	–0.145	–0.275	–0.016	0.023
Fig. 6C	VGLUT1/VGAT puncta density	CA1	TS EE-EGCG_TS	Continuous variable	Mixed-model *F*-test	–0.245	–0.442	–0.047	0.01
Not shown	VGAT puncta size	CA1	WT EE-EGCG_WT	Continuous variable	Mixed-model *F*-test	0.049	–0.003	0.102	0.07
Fig. 6D	VGLUT1/VGAT % of area	CA1	WT EE-EGCG_WT	Continuous variable	Mixed-model *F*-test	–0.137	–0.267	–0.007	0.035

In CA1, Ts65Dn mice also showed significantly increased density of VGLUT1 puncta of reduced size compared with WT mice (data not shown; Table 6). As in DG, Ts65Dn mice presented no differences in VGAT density puncta in CA1, but in this region, VGAT puncta were enlarged (data not shown; Table 6). This resulted in an increased ratio of VGLUT1/VGAT puncta density (Fig. 6*C*; Table 6), and a reduced VGLUT1/VGAT percentage of area occupied (Fig. 6*D*; Table 6).

Compared with untreated conditions, EE-EGCG–treated Ts65Dn mice exhibited a significant reduction in the density of VGLUT1 puncta in DG (data not shown; Table 6) but not in CA1. VGLUT1 puncta were significantly enlarged in DG (data not shown; Table 6) but not in CA1. There were no significant differences in VGAT puncta number or size between EE-EGCG–treated and untreated Ts65Dn mice in DG or CA1. As a result, EE-EGCG–treated Ts65Dn mice showed a reduction in the ratio of VGLUT1/VGAT density in both DG (Fig. 6*A*; Table 6) and CA1 (Fig. 6*C*; Table 6), leading to values that were similar to those of WT mice. On the other hand, EE-EGCG–treated WT mice also showed a trend toward an enlargement of VGAT puncta size in CA1 (data not shown; Table 6), leading to a decreased VGLUT1/VGAT percentage of area occupied (Fig. 6*D*; Table 6) in comparison to untreated WT mice.

## Discussion

In the present study, combined EE-EGCG treatment significantly increased spine density in CA1, normalized excitatory and inhibitory synaptic markers in CA1 and DG, and improved performance in a corticohippocampal-dependent learning task in young Ts65Dn mice.

In line with previous studies, we detected poor learning strategies and hippocampal-dependent learning and memory performance in the MWM in young Ts65Dn mice (Escorihuela et al., 1995; Reeves et al., 1995). In Ts65Dn mice, but not WT mice, EE-EGCG treatment improved performance in the MWM, reducing escape latency and Gallagher index and distance during the learning sessions. PCA confirmed that untreated Ts65Dn showed inefficient learning progress over acquisition sessions, reaching maximum values of PC1, a global learning variable, similar to initial WT values. EE-EGCG–treated Ts65Dn mice improved on global learning measures. They reached more advanced maximum PC1 values than untreated Ts65Dn mice, suggesting a modification in learning-related behavior as previously reported in middle-age Ts65Dn mice (Catuara-Solarz et al., 2015).

Ts65Dn mice also exhibited poor reference memory, as indicated by a significantly increased Gallagher index and distance and latency to the first entry to the platform area in the probe trial. However, no genotype effects were detected in other variables, such as time spent in the target quadrant or latency to first entry to target area, probably because of the high variability of the data. Additionally, Ts65Dn mice presented a deficit in cognitive flexibility, as shown by the inefficient performance during the reversal sessions (executive function).

Single-variate analysis of different parameters between EE-EGCG–treated and untreated Ts65Dn mice did not reach statistical significance at reference memory and reversal sessions. However, a significant enhancement in cognitive flexibility was shown by multivariate analysis of the reversal sessions. In middle-age Ts65Dn mice, EE-EGCG treatment improved learning and reference memory, but not cognitive flexibility (Catuara-Solarz et al., 2015). This suggests that EE-EGCG treatment effects depend on age and cognitive domain, possibly because of differential effects on different underlying brain regions and functions at different ages. EE-EGCG–treated WT mice did not show significant differences, possibly because of a ceiling effect.

MWM is a learning paradigm based on the stressful and aversive stimuli of the water pool, which triggers increases in plasma corticosterone, leading to a motivational state in the mice to learn the spatial configuration of the cues to escape (Harrison et al., 2009). Previous studies have shown that EGCG exerts an anxiolytic effect on different behavioral anxiety tests such as the forced swimming test, elevated plus maze, passive avoidance test, and tail suspension test (Dias et al., 2012). It could thus be speculated that the potential anxiolytic effects of EGCG would contribute to the learning improvement we found in the MWM. However, a number of facts suggest that the learning improvements found in treated Ts65Dn mice are not mainly contributed by the anxiolytic effect of EGCG. Ts65Dn mice have reduced levels of anxiety-like behavior in the elevated plus maze (Coussons-Read, 1996; Demas, 1996; Escorihuela, 1998; Shichiri, 2011), suggesting that the learning deficits shown by Ts65Dn in the MWM are not associated with anxiety. Thus a potential anxiolytic effect of EGCG would not eventually lead to significant learning improvement. Additionally, in the case of a potential MWM improvement associated with the anxiolytic effect of EGCG, we should be able to observe it in the control group that is subjected to the same anxiogenic scenario. However, in our study, the WT group did not benefit from the combined EE-EGCG treatment.

Even so, we also addressed the effects of the combined EE-EGCG treatment in a less stressful learning test, the novel object recognition test. The performance of this test depends on the functionality of the entorhinal and perirhinal cortices and the hippocampus (Brown and Aggleton, 2001; Brown et al., 2010). In this test, trisomic mice presented no significant deficit in their DI in comparison to WT mice, similar to some previous reports (Hyde and Crnic, 2002), although a slight tendency to impairment was detected that is in line with data from Fernandez et al. (2007). EE-EGCG–treated Ts65Dn mice presented an improvement in DI with respect to untreated counterparts and scored at similar levels to WT mice. On the other hand, EE-EGCG–treated WT mice showed a poorer performance than untreated WT mice, suggesting a possible deleterious effect of EGCG.

Along with learning improvement, EE-EGCG–treated mice showed significant neuromorphologic changes in the hippocampus. Consistent with previous reports in DS (Ferrer and Gullotta, 1990) and Ts65Dn mice (Belichenko et al., 2004), we observed a reduction in dendritic spine density in outer molecular layer dendrites from granule cells of the DG, and in apical dendrites of pyramidal neurons of CA1 in Ts65Dn mice. Combined treatment with EE-EGCG partially rescued the dendritic spine density deficit in CA1, but not in the DG of Ts65Dn mice. A reduction of Dyrk1A kinase activity (Bain et al., 2003; Golabek et al., 2011; Pons-Espinal et al., 2013), but also other signaling pathways that are modified by both EE and EGCG, such as increased CREB and Akt phosphorylation (Jia et al., 2013; Ramírez-Rodríguez et al., 2014; Ortiz-López et al., 2016) or increases in BDNF expression (Young et al., 1999; Li et al., 2009a, 2009b), could contribute to these neuroplasticity changes.

We also explored the effects of EE-EGCG treatment on E/I balance, using excitatory (VGLUT1) and inhibitory (VGAT) synaptic vesicle markers. In Ts65Dn DG, VGLUT1 puncta were more abundant but smaller, with no changes in the percentage of area occupied, whereas VGAT puncta showed no differences compared with WT littermates. In CA1, Ts65Dn mice showed the same phenotype, VGLUT1 puncta being more abundant and smaller; however, in this region, VGAT puncta were enlarged, leading to an increased VGLUT1/VGAT ratio but a reduction of VGLUT1/VGAT percentage of area occupied. That in both DG and CA1 VGLUT1 puncta were more abundant and smaller in Ts65Dn could affect the probability or efficiency in neurotransmitter release (Harris and Sultan, 1995; Bozdagi et al., 2000; Antonova et al., 2001; Bamji et al., 2006; Bourne et al., 2013) and could also be related to the previously reported enhanced GABA_A_ and GABA_B_ evoked inhibitory postsynaptic currents in DG of 3- to 4-month-old male mice (Kleschevnikov et al., 2012) and increased GABA release in the hippocampus of male and female adult mice (Begenisic et al., 2011). Consistent with our results, previous work showed no changes in density of VGAT puncta nor density of inhibitory synapses using electron microscopy in the DG of 3-month-old male mice, although apposition length of symmetric (inhibitory) synapses was larger (Belichenko et al., 2009). Additionally, Kleschevnikov et al. (2012) found no differences in GAD67 optical density in DG, with only a trend toward reduction in GAD67 in the outer molecular layer of 3- to 4-month-old male mice. A recent study using Western blot showed a reduction in the hippocampal expression of VGLUT1 and no statistical difference in VGAT (Souchet et al., 2015). Conversely, others have shown an increase in the percentage of area occupied by VGAT puncta and VGAT/gephyrin puncta in DG of 4.5- to 5.5-month-old male mice (Martinez-Cue et al., 2013). Differences in experimental methods, hippocampal subregions, age, or sex could account for these divergent results. On the other hand, very little is known about how E/I is affected across different brain regions or ages in Ts65Dn mice. Possibly, the E/I imbalance could arise from alterations in excitation, inhibition, or both and may be continuously changing as a result of synaptic plasticity, leading to region-specific dysfunction (Bartley et al., 2015).

Interestingly, EE-EGCG–treated Ts65Dn mice showed normal density and size of VGLUT1 puncta, and as a consequence, the balance of excitatory and inhibitory puncta in DG and in CA1 was also in the normal ranges. That the treatment restores the density and size of VGLUT1 puncta is consistent with the treatment effect on the density of dendritic spines. Excitatory synapses comprise a presynaptic terminal with abundant synaptic vesicles containing glutamate, in association with dendritic spine heads acting as a postsynaptic element. Thus, our results suggest that combined treatment with EE-EGCG may increase excitatory synaptic connections.

These results are also consistent with the outcome of a recent phase II clinical trial with DS individuals in which a therapy combining cognitive training and EGCG normalized neuronal network functionality, as measured by functional MRI, and cortical excitability by transcranial magnetic stimulation (de la Torre et al., 2016).

Conversely, in WT mice EE-EGCG treatment reduced spine density in CA1, but not in the DG, and led to E/I imbalance in CA1, without significant changes in the DG. This deleterious effect could be explained by an overinhibition of Dyrk1A kinase activity in WT conditions, since it has been shown that *DYRK1A* haploinsufficiency is associated with neuroanatomical and neuroarchitectural defects in flies, mice, and humans. Indeed, mutant flies with reduced Dyrk1A expression present reductions in the volumes of the adult optic lobes and central brain hemispheres (Tejedor et al., 1995). Brains from Dyrk1A heterozygous mice are ∼30% smaller and have reduced size and weight in specific brain regions, along with reduced neuronal density in the superior colliculus and increased neuronal numbers in brain regions such as somatosensory and motor cortices (Fotaki et al., 2002), with significantly smaller and less complex basal dendritic arbors and reduced dendritic spine densities (Benavides-Piccione et al., 2005). In the hippocampus, Dyrk1A heterozygous mice show a significant reduction in hippocampal thickness, accompanied by decreases in cell number in CA1, CA2, CA3, and DG (Arqué et al., 2008). Humans with *de novo* heterozygous variants of DYRK1A also have congenital microcephaly, structural brain abnormalities, and intellectual disability (Møller et al., 2008; Ji et al., 2015).

Taken together, our results suggest that combined treatment with EE and EGCG is a potent cognitive enhancing intervention for DS. We demonstrated that EE-EGCG treatment–derived cognitive improvements are associated with neuromodulatory effects at the hippocampus that normalize defects in dendritic spine density and E/I synaptic puncta ratio. Overall results suggest that combined EE-EGCG treatment has the capacity to simultaneously target several abnormal processes underlying intellectual disability in DS which would be optimal for a disease-modifying intervention in this clinical population.

## Synthesis

The decision was a result of the Reviewing Editor Carmen Sandi and the peer reviewers coming together and discussing their recommendations until a consensus was reached. A fact-based synthesis statement explaining their decision and outlining what is needed to prepare a revision is listed below. The following reviewers agreed to reveal their identity: Juan Nácher, Mark Good

After discussing your manuscript with the two reviewers, we have agreed that the work is very interesting article, as it sheds light on the neurobiological bases of cognitive deficits in Down Syndrome (DS) and those of a promising new therapy. More specifically, the work re-evaluates, in a DS animal model, the beneficial effects of a treatment with epigallocatechin-3-gallate and cognitive stimulation already reported to be beneficial in young adult DS patients. The study recapitulates the cognitive improvement measuring hippocampal dependent learning/memory, induction of dendritic spine remodeling in CA1 pyramidal neurons and restoration of the excitatory/inhibitory balance in CA1 and dentate gyrus. Overall, we consider that the study is well conducted, with an excellent design and the article is very well written and organized, and the graphs and tables are of good quality (although some microphotographs are missing).

Our major concerns are the unconventional statistics used and the low effect size of the treatment, particularly in regards to the behavioral data:

-In fact, our impression is that the TS treatment group show almost no improvement in latency by the end of training. We suspect that a standard ANOVA would reveal no overall interaction or differences. We would like to ask the authors to either explain why these statistics are valid in a convincing manner or to reconsider the use of more traditional statistical approaches and modify the conclusions of the study to emphasize the non-behavioral data while acknowledging that the behavioral data would not be significant with traditional statistical analyses.

-Is it the case that the Gallagher measure is independent of the propensity to show thigmotaxis in the TS and TS treated groups? Note that the Gallagher measure does show a small difference between the TS and TS treated groups, but again it does not appear to be any change as a function of training in either the TS or TS treated groups. Performance on session 5 looks very similar to that achieved on session 1 for both groups.

-The data from the probe trial should be included, particularly to validate the behavioral study as to the behavior displayed by the control group.

-Please, provide information about when the cued trial was conducted, whether at the start or end of the spatial training stage.

-Although the discussion leaves one with the impression that enrichment and epigallocatechin treatment ameliorated the TS cognitive deficit, there are perhaps grounds to be more cautious. For example, epigallocatechin treatment is known to interact with anxiety. Could an interaction of epigallocatechin with water maze-induced anxiety be a contributing factor to the "performance" changes observed in this study.

-The study would have benefited from an additional behavioural test that used different visuo-spatial, motoric and motivational requirements from the water maze. This would establish the generality of any "learning" improvement across sensory domains and task requirements (e.g., object novelty detection). At a minimum, some mention of this point in the discussion would be worthwhile given the variability in response to the EE and epigallocatechin treatment between studies, which the authors also discuss.

We also have several important concerns regarding the structural studies, as follows:

-The ms. should clearly state that the n used for the spine analysis was the number of dendrites and not the number of animals.

-The ms. should also indicate that the spine density analysis has been performed in the APICAL dendrites of the DG granule neurons, the length of the dendritic segment in which analyses were performed and its distance to the soma. In line 434 (discussion) it is erroneously indicated that the basal dendrites of granule neurons have been analyzed.

-Whenever still possible within the existing preparations, it would be very informative if an analysis of the different types of spines (mushroom, thin, stubby) can be performed. If this is not possible, please, indicate in the text the criteria used to identify a spine.

-The ms. should indicate from which regions (specific layers) the images for I/E balance analysis were taken. What was the surface covered by these photographs? How many images were taken per region/layer? Were they taken randomly?

-Analyses were performed only on 4 animals per group which we consider too low. Please, either explain based on a power analysis why this is an acceptable "n" or, if needed to satisfy requirements from the power analyses, please add some extra animals to the study.

Finally, we have the following minor comments and suggestions:

-Line 377, substitute: "not show statistically significant differences in DG dendritic spine density neither in Ts65Dn nor in WT mice" by "not show statistically significant differences in DG dendritic spine density neither with Ts65Dn nor with WT mice"

-It would be helpful for the reader to have a little paragraph discussing the putative relationship between that increase in spine density elicited by the treatment and the parallel changes in E/I balance.

-Please include at least the title of the De la Torre, Lancet Neurology paper indicated as "in press", in the reference list.

-Please provide in the discussion (line 480) more details on the neuroanatomical and neuro-architectural defects found in DYRK1A haploinsufficiency.

-Although the authors have explained convincingly why they have used females, it would be helpful for the reader to know that the majority of females from most inbred strains first ovulate naturally between 6 and 8 weeks after birth, so it is unlikely that variations in estrogen levels between females could influence behavior, spine density or E/I balance.

-The material and methods and results sections should indicate that authors measured dendritic spine density on basal dendrites of CA1 pyramidal neurons. They also should indicate the length of the dendritic segment in which they are performing the analyses and its distance to the soma.

-Please include at least the title of the De la Torre, Lancet Neurology paper indicated as "in press", in the reference list.

-Please provide in the discussion (line 480) more details on the neuroanatomical and neuro-architectural defects found in DYRK1A haploinsufficiency.

-The journal name is missing in Altafaj et al. Reference.
